# Assessment of tuberculosis spatial hotspot areas in Antananarivo, Madagascar, by combining spatial analysis and genotyping

**DOI:** 10.1186/s12879-017-2653-9

**Published:** 2017-08-14

**Authors:** Noël Harijaona Ratovonirina, Niaina Rakotosamimanana, Solohery Lalaina Razafimahatratra, Mamy Serge Raherison, Guislaine Refrégier, Christophe Sola, Fanjasoa Rakotomanana, Voahangy Rasolofo Razanamparany

**Affiliations:** 1 Unité des Mycobactéries, Institut Pasteur de Madagascar, Antananarivo, Madagascar; 2 Institute for Integrative Biology of the Cell (I2BC), CEA, CNRS, Univ. Paris-Sud, Université Paris-Saclay, 91198 Gif-sur-Yvette cedex, France; 3 Programme National de Lutte contre la Tuberculose (PNLT), Ministère de la Santé, Antananarivo, Madagascar; 4 Unité d’Epidémiologie, Institut Pasteur de Madagascar, Antananarivo, Madagascar

**Keywords:** *Mycobacterium tuberculosis*, Geographic Information System, Genotyping, Spatial cluster, Antananarivo

## Abstract

**Background:**

Tuberculosis (TB) remains a public health problem in Madagascar. A crucial element of TB control is the development of an easy and rapid method for the orientation of TB control strategies in the country. Our main objective was to develop a TB spatial hotspot identification method by combining spatial analysis and TB genotyping method in Antananarivo.

**Methods:**

Sputa of new pulmonary TB cases from 20 TB diagnosis and treatment centers (DTCs) in Antananarivo were collected from August 2013 to May 2014 for culture. *Mycobacterium tuberculosis* complex (MTBC) clinical isolates were typed by spoligotyping on a Luminex® 200 platform. All TB patients were respectively localized according to their neighborhood residence and the spatial distribution of all pulmonary TB patients and patients with genotypic clustered isolates were scanned respectively by the Kulldorff spatial scanning method for identification of significant spatial clustering. Areas exhibiting spatial clustering of patients with genotypic clustered isolates were considered as hotspot TB areas for transmission.

**Results:**

Overall, 467 new cases were included in the study, and 394 spoligotypes were obtained (84.4%). New TB cases were distributed in 133 of the 192 Fokontany (administrative neighborhoods) of Antananarivo (1 to 15 clinical patients per Fokontany) and patients with genotypic clustered isolates were distributed in 127 of the 192 Fokontany (1 to 13 per Fokontany). A single spatial focal point of epidemics was detected when ignoring genotypic data (*p* = 0.039). One Fokontany of this focal point and three additional ones were detected to be spatially clustered when taking genotypes into account (*p <* 0.05). These four areas were declared potential TB transmission hotspots in Antananarivo and will be considered as priority targets for surveillance in the future.

**Conclusion:**

This method, combining spatial analysis and TB genotyping will now be used for further focused clinical and epidemiological studies in Madagascar and will allow better TB control strategies by public health authorities.

**Electronic supplementary material:**

The online version of this article (doi:10.1186/s12879-017-2653-9) contains supplementary material, which is available to authorized users.

## Background

Tuberculosis (TB), caused by *Mycobacterium tuberculosis* complex *(M. tuberculosis)*, remains one of the deadliest infectious diseases worldwide. In 2014, 9.6 million people contracted TB and 1.5 million died from the disease [1]. The number of deaths due to TB slowly declined between 2000 and 2013 due to effective diagnosis and treatment, but remains unacceptably high [2].

Spoligotyping is a method investigating the diversity at a highly variable CRISPR locus evolving by deletion in *M. tuberculosis* complex [3]. Spoligotyping has been widely used to classify *M. tuberculosis* clinical isolates by family and subfamily [4, 5] that were later found to be largely concordant with lineages as defined by an whole genome sequencing (WGS) approach, allowing single nucleotide polymorphisms (SNPs) to be identified [6]. In addition and despite a relatively low discrimination level of spoligotyping [7, 8] it can be used for first genetic identification of patient’s clinical isolates and suggest or exclude recent transmission cases which should be investigated further using more discriminatory methods [7, 9, 10]. Previous studies demonstrated that spatial clustering of TB data when associated to genetic clustering of TB cases more easily allows to focus on adequate settings to distinguish most vulnerable populations and reactivation versus recent transmission cases [10–13].

Hence, geospatial tools may be helpful to study the TB dynamics of urban areas with high prevalence of TB. Geospatial tools incorporating Geographic Information Systems (GIS) enable the identification and mapping of spatiotemporal clustering of disease or patients [14]. The GIS method has been used to study the spatial distribution of human TB cases and has identified the heterogeneity of epidemic areas [10, 15]. To confirm recent TB transmission, isolates must be found to be clonal. Clonality must then be investigated by more discriminatory genotyping methods. In Antananarivo, an exhaustive thorough genomic characterization of clinical isolates either by 24 MIRU-VNTR or by WGS remains out of reach for economic reasons for the time-being. For this reason, we chose a classical spoligotyping approach, which remains a first-line method to characterize clinical isolates in resource-limited countries. Such a combination of approaches (spatial and genetic clustering) is interesting to locate spatial clusters of TB and attempt to assess where recent TB transmission cases may occur.

In Madagascar, the incidence of TB in 2013 was estimated to be approximately 233/100,000 inhabitants [16]. TB prevalence distribution in Madagascar is likely heterogeneous with particularly high rates in specific areas driven by uncontrolled transmission, as in most resource-limited countries with either remote or isolated settings [15, 17, 18]. A crucial element of TB control efforts is the identification of “TB Hotspot areas” for the orientation of TB control strategies given the lack of resources. Therefore it would be beneficial for the TB program to have a tool for targeting areas of high transmission risk where interventions should be concentrated. Previous studies using genotyping techniques on clinical isolates have shown a large diversity of circulating *M. tuberculosis* genotypes in Madagascar [19–21]. A preliminary study based on TB notification rates identified spatial aggregation of TB cases in Antananarivo [15, 17]. This aggregation could be due either to actual transmission and/or to reactivation cases. Our aim here was to accumulate evidence concerning the possibility that previously identified TB hotspot areas in Antananarivo could be linked to transmission events.

We used a combination of spatial tools and genotyping to identify potential high-prevalence and likely higher recent transmission risk, TB areas in Antananarivo, Madagascar, as done recently in Brazil and Japan [11, 12]. These methods will be used in Madagascar for further understanding of the TB epidemiology in Antananarivo and the identification of priority targets for TB control strategies.

## Methods

### Study area

**Fig. 1:**
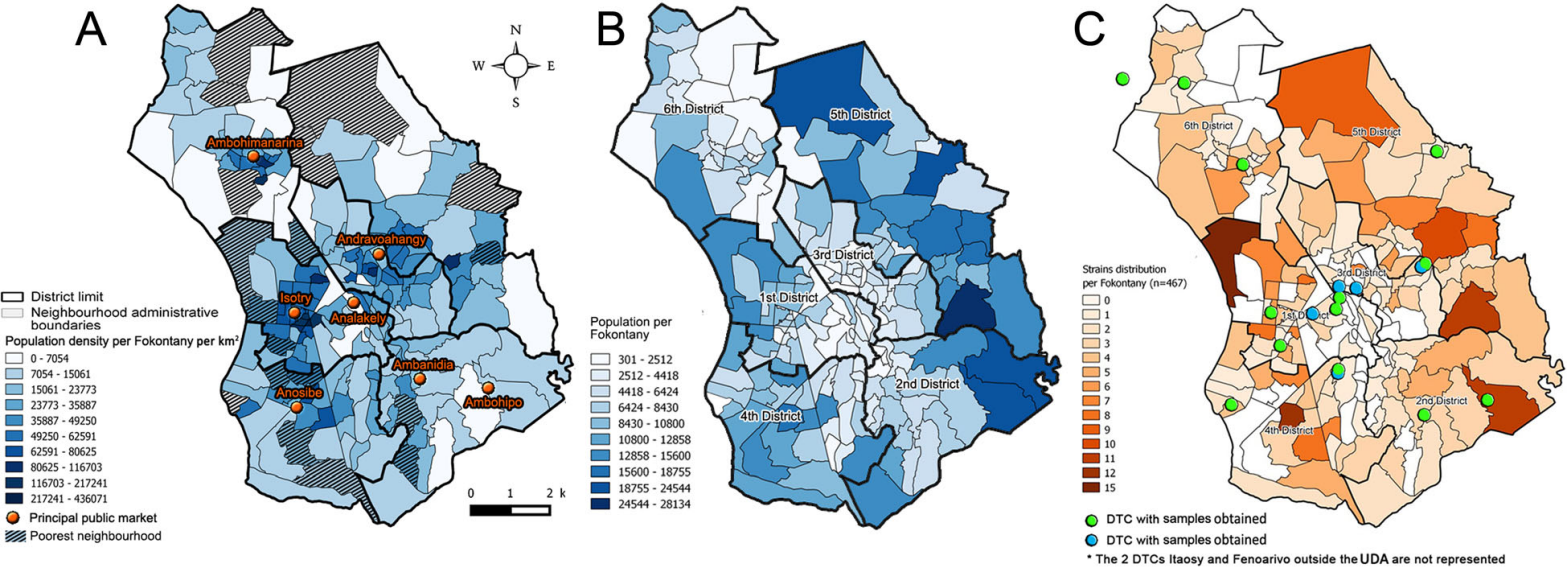
Structure of the urban district of Antananarivo (UDA). **a** describes the distribution of population density per Fokontany and the localization of the principal public markets in the UDA. The poorest Fokontany are hatched. **b** describes the distribution of the most populous Fokontany. **c** describes the distribution of TB cases recruited for the study. TB diagnosis and treatment centers included in the study where represented in *green* or *blue point*

The study was carried out in Antananarivo, the capital of Madagascar (surface area = 90 km^2^; 1.1 million inhabitants). Antananarivo is divided into six districts and sub-divided into 192 “Fokontany” that are administrative neighborhoods. Population density per Fokontany and population size is highly heterogeneous, as in many large cities (Fig. 1a and b); the poorest neighborhoods were classified by average household income; the 25% of all neighborhoods with the lowest average income were assigned as “poorest neighborhoods”; the most populous and poorest neighborhoods are localized in the periphery (Fig. 1a and b) [22]. Some public markets are distributed in the densest zones (Fig. 1a). There is circulation of poorest and most vulnerable populations in these public markets. The first district is among the poorest and most populous in Antananarivo. Cases were diagnosed and treated in TB diagnosis and treatment centers (DTCs) indicated in Fig. 1c.

Fokontany were identified by conventional alphanumeric codes and were geolocalized by their centroid. Data of the population census of the 192 Fokontany were provided by the Development Office of Antananarivo (DOA) and poorest neighborhoods were described previously [22].

### Study population

New confirmed TB cases for individuals were included without age limitation between August 2013 and May 2014 (*n* = 523). Only patients living in Antananarivo for at least one year have been recruited since they are more likely to have got TB in this study area. All 17 DTCs in the study area and three reference DTCs in the suburban zone of Antananarivo (Fenoarivo, Ambohibao, and Itaosy), totaling 20 DTCs were included in the study. The DTC of the central jail was excluded because of the potential transmission bias associated with this last area.

For each recruited patient, the objectives and nature of the study were explained by a DTC agent. Information written in Malagasy language was given to any patient or parents for children under 18 years. He/she was then asked if he/she agrees to participate in the study. Study subjects were allowed to refuse to participate in the study or withdraw from the study at any time without prior justification. When the subject agreed to participate in the study, he/she signed the informed consent form. An interview was conducted and the information was collected on a form. The residence address and Fokontany were recorded for every patient. The residence address provided by the patients in the questionnaire was confirmed by the DTC registers.

### Genotyping

Fresh sputa were collected, stored, and cultured on Lowenstein Jensen (LJ) solid medium. Raw DNA was obtained from the clinical isolates by heating and killing a suspension of a culture colony in a hot dry bath at 80 °C for 30 min, After centrifugation at 13,000 * G for 5 min, supernatant was transferred in a new tube and conserved at −20 °C. Raw DNA was shipped at −20 °C to Orsay, France where the spoligotyping was performed directly using a Luminex 200 platform as described by Zhang [23]. The spoligotype profiles were identified by their Shared-type (SIT – Spoligotyping International Types) and lineage designation by using the SpolDB4/SITVITWEB rules and classification [4]. Moreover, to fit with current genome-based lineage designations, L1-L7 lineage labels were added to spoligotype families [24, 25]. Given the modest benefit of spoligotyping value when used alone in molecular epidemiological studies, we did not infer any recent transmission rate based on spoligotyping-based clustering, but used the genotyping information to correlate spatial and genetic clustering as a clue to define “Hot Spot TB area”.

### Spatial analysis

TB patients were localized according to their Fokontany of residence. All data on the population denominators per Fokontany and metadata used for mapping were provided by the DOA. All TB patients and patients with genotypic clustered isolates (patients associated with isolates with repeated spoligotype or PRS) were scanned separately using the Kulldorff spatial scanning method. The populations per Fokontany with SaTScan™ were considered for the spatial analyses (http://www.satscan.org) [26]. For Satscan® parameters, a maximum radius of 1 km was defined for the spatial scanning. A *p-*value <0.05 determined by conducting Monte Carlo replications was considered to be statistically significant. We assumed that the number of TB patients in each Fokontany fits with the Poisson distribution. The identified Fokontany associated with spatial clusters representing TB focal points of epidemics were mapped using QuantumGis 2.8® (QGIS Development Team, 2013). Areas representing focal points of epidemics with highly genetically related isolates (clustered by spoligotyping) were considered to be potential TB hotspot transmission areas.

## Results

### Study participants

**Fig. 2:**
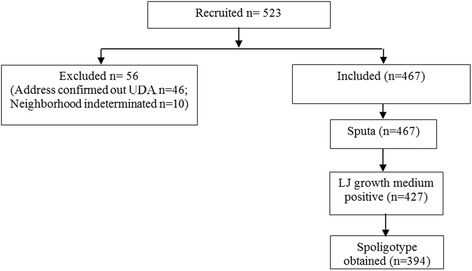
Quantitative data on the studied sample population. Out of a population of 1.1 million people investigated during 8 months, and given the previous estimated TB incidence rate in Antananarivo (141 cases/100000 inhabitants/year), we reached a 25% exhaustivity in our recruitment and a 86% of genotyping rate success once geographically inadequate cases were excluded

A total of 523 TB patients were recruited from 14 of the 20 DTCs included in the study. The recruited patients were localized to 142 of the 192 Fokontany of Antananarivo (1–15 patients/Fokontany). Fifty-six patients were excluded (the addresses of 46 patients were confirmed outside the study area and residence’s Fokontany of 10 patients could not be identified). Among the 467 remaining included patients, 427 (91.4%) had positive bacterial cultures identified from the LJ-growth media (Fig. 2).

### Genotypic diversity of the clinical isolates

We obtained 88 individual spoligotype profiles from 394 clinical isolates (84.4% of the 467 recruited patients). We could not type 73 (16%) clinical isolates as 40 cultures were negative and 33 spoligotypes were non-interpretable. Of the individual 88 spoligotype profiles, 47 were unique and 41 were clustered, totaling 347 clustered isolates, found in clusters of 2 to 39 clinical isolates (Additional file 1: Table S1).

**Fig. 3:**
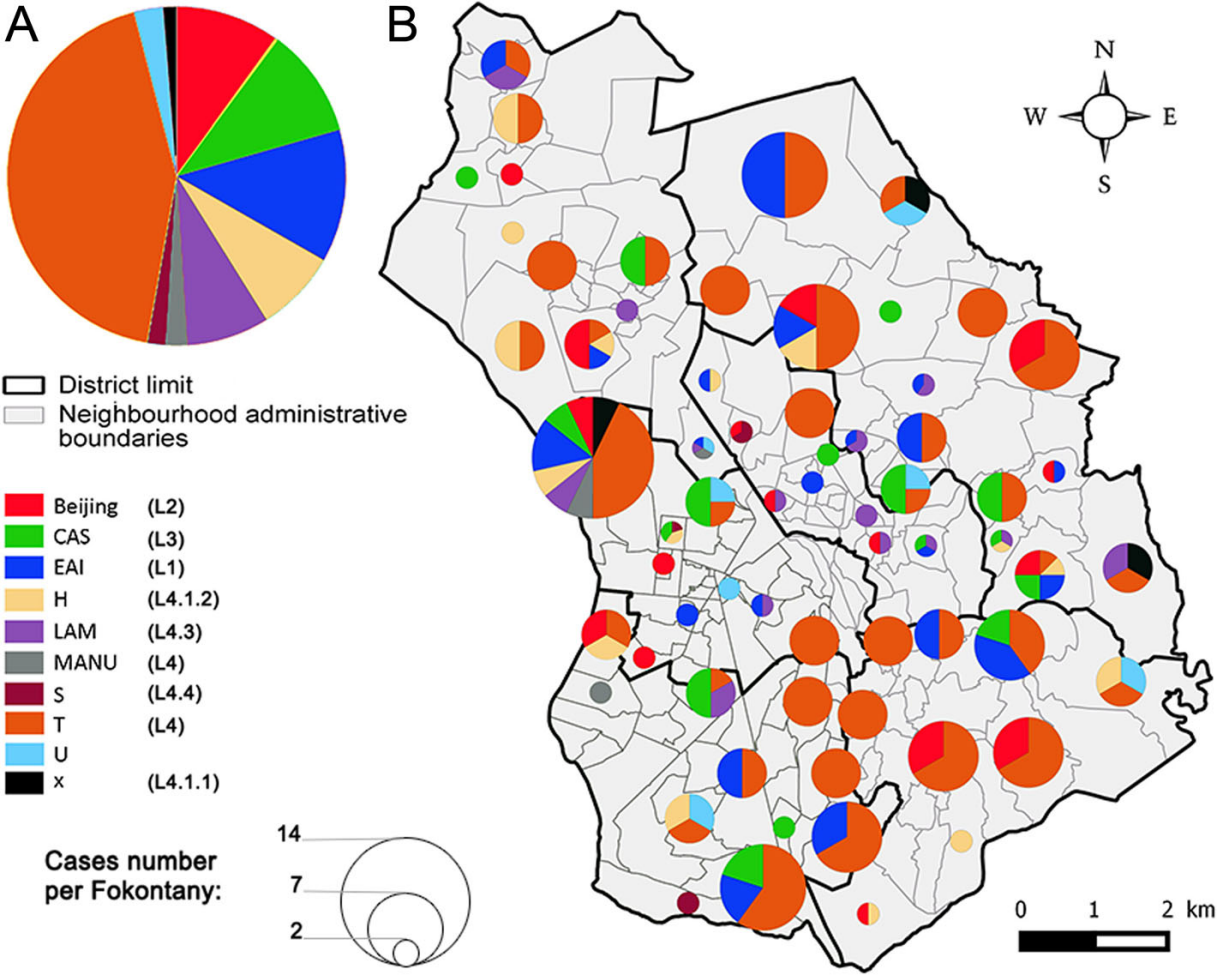
**a** Distribution of spoligotyping-defined lineages obtained on 394 spoligotypes: spoligotyping nomenclature according to Brudey et al. [5], followed by genome-based (L1 to L4) lineage and sublineage nomenclature according to Coll et al. [24]. **b** Spatial distribution of *M. tuberculosis* spoligotyping-defined lineages within the 192 Fokontany of Antananarivo. (size of circle is proportional to cases number, cf. Figure)

Clinical isolates belonging to the Euro-American/T spoligotyping family (included in Lineage 4; 43.1%) predominated, followed by the EAI lineage (Lineage 1; 12.7%), and the CAS lineage (Lineage 3; 10.4%), whereas the Beijing lineage (Lineage 2), the LAM, and the H sublineages (both belonging to Lineage 4) did not exceed 10% of the studied clinical isolates (Fig. 3). The 14 most prevalent SITs constituted 70.0% of all spoligotypes (276/394). Most prevalent SITs (more than 30 cases) were: SIT86 (T family), SIT1 (Beijing lineage), SIT156 (T family), SIT109 (EAI lineage), SIT21 (CAS lineage) (Additional file 1: Table S1).

### Spatial analysis

Residences of the included patients (*n* = 467) were distributed in 133 of the 192 Fokontany (Fig. 1c). Spatial observations showed that the highest number of TB cases was observed in Fokontany Andohatapenaka II in the 1st district (*n* = 15) whereas the district with the highest number of TB cases was the 5th district (112/467; 24.0%). The relatively most populous Fokontany at the periphery of Antananarivo had the higher TB cases number compared to the center of the city (3rd district) (Fig. 1b, c).

**Fig. 4:**
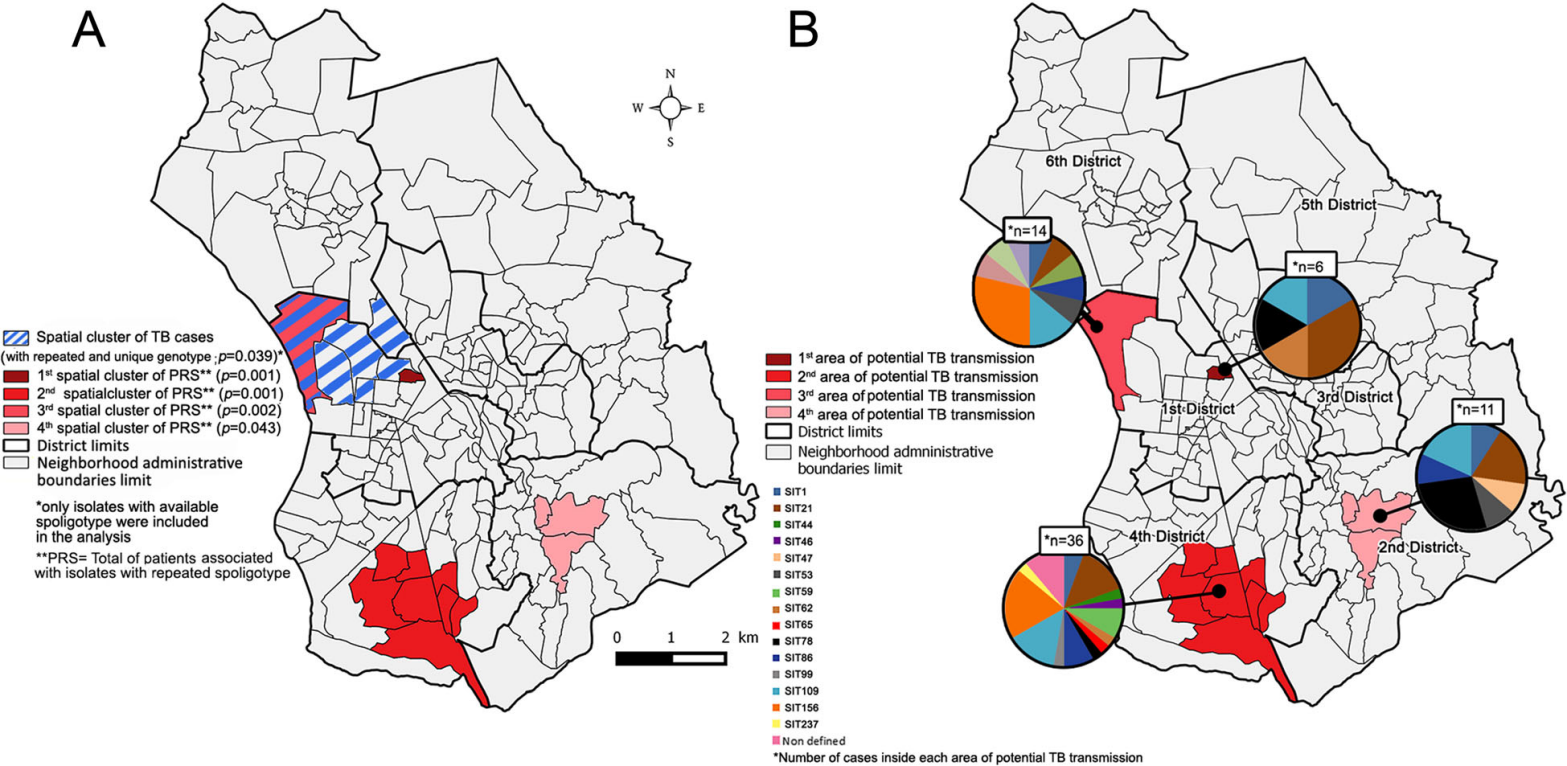
Spatial signatures of TB identified by the Kulldorff spatial scan method. **a** Spatial clustering of TB cases and patients with genotypic clustered isolate. **b** distribution of isolates families in each spatial clustering of patients with genotypic clustered isolates

With the spatial scanning method of Kulldorff, we observed one significant spatial cluster of TB cases (*p* = 0.039) formed by 6 Fokontany (localized in the 1st and the 6th neighboring districts) (Fig. 4a). The 394 patients with genotyped clinical isolates were distributed in 133 Fokontany, The patients with genotypic clustered isolates (*n* = 347) were distributed in 127 of these 133 Fokontany (1 to 13 isolates per Fokontany); and when scanning these 347 patients, four significant spatial clusters were observed (Fig. 4a): a first cluster formed by one Fokontany (Antohomadinika Afovoany) in the 1st District (*p* = 0.001), a second formed by eight Fokontany of the 4th District (*p* = 0.001), a third cluster in one Fokontany (Andohatapenaka II) in the 1st district (*p* = 0.002) which overlapped with the spatial cluster of all TB cases (Fig. 4a), and a 4th cluster formed by three Fokontany in the 2nd district (*p* = 0.043). These spatial clusters represent potential areas of TB transmission. The spoligotype diversity in each spatial clustering is presented in Fig. 4b. The first area with potential TB transmission included two SIT21 isolates. There were seven SIT156 isolates in the second area, followed by SIT21 (*n* = 5) and SIT109 (*n* = 5). SIT156 (*n* = 4) and SIT109 (*n* = 2) predominated in the 3rd area with potential TB transmission and SIT78 (*n* = 3) and SIT59 (*n* = 3) predominated in the 4th area (Fig. 4b). These TB hotspots could not be detected on the spatial distribution of the 11 lineages (Fig. 3b).

## Discussion

This study aimed to determine the spatial signature of TB by using a combination of genotyping and geospatial tools across the urban city of Antananarivo, Madagascar. The complementarity of both the genotyping and spatial analyses approaches has been used for the determination of TB transmission areas and risk studies in Brazil and Japan [11, 12]. These two methods are not new but they had never been combined for the detection of potential high risk areas of TB transmission in Antananarivo.

The combination of GIS and spoligotype data identified four hotspots with potential TB transmission in the city of Antananarivo. Analysis of all TB cases (without distinction of genotypic clustering of strains) identified a TB disease focal point overlapping with one of the potential transmission area (constituted by the Fokontany of Andohatapenaka II). TB hotspots such as that detected by the analysis taking only spatial data into account is likely constituted simultaneously by patients linked by indirect and relatively ancient TB transmission and by patients linked by local and recent TB transmission. Spatial clustering of patients with genotypically clustered isolates may therefore concern more patients associated with local and recent transmission. Additionally, our combination of approaches permitted the detection of spatial clusters of TB patients which were not detected with only the spatial scan of TB cases. TB transmissions might have occurred in these areas. For definitive proof of TB transmission, more discriminatory genotyping tools should however be used.

While previous studies determined risk factors associated with spatial clustering of TB cases in Antananarivo [15, 18], risk factors associated with transmission were poorly investigated. The first potential transmission hotspot is constituted by the Fokontany of Antohomadinika Afovoany that is localized in one of the poorest neighborhood in the 1st urban district of Antananarivo. Most of the houses in this area are made of precarious wooden hovels and the majority of the local population does not have standard health care access. While life under fragile conditions and environmental factors [14, 19] are known factors that contribute to TB reactivation [16], they could also foster local transmission as suggested by this study. The relatively high diversity of SITs seen in this poor area supports the previous findings that environmental factors also favor reactivation of latent TB. This area, containing the highest rate of TB, constitutes the first high risk area of TB transmission in Antananarivo.

The three other areas of potential TB transmission (#2, #3 and #4) host or lie close to three public markets (Anosibe, Isotry and Ambanidia market) where there is an important flow of people. Similar studies have shown that a flow of persons promotes TB transmission [11, 12]. This study further adds evidence for potential transmission in markets.

The discriminatory level of spoligotyping is relatively low, and the genotypic clustering cannot be used to estimate TB recent transmission rates. Thus isolates with the same spoligotype may be coming from different chains of transmission. However, when compared with isolates with single genotype, that might represent reactivation or relatively far recent transmission, these clustered isolates are more likely to have originated from recent spatially localized transmission. The aim of the study was to identify high risk areas, this limitation does therefore not invalidate this study and should be taken as a first step towards identification of real TB transmission hotspots.

From 1994 until 2000, studies of the *M. tuberculosis* genotype profiles present in Antananarivo reported six lineages and sub-lineages of *M. tuberculosis* clinical isolates: T, LAM, and H (Lineage 4), EAI (Lineage 1), CAS (Lineage 3), and Beijing (Lineage 2) [19]. The 6 lineages were shown to be present in the capital of Madagascar in 2004/2005, with the appearance of other minor sub-lineages such as S and X along with some unknowns (U) [27]. The distribution of the major circulating clinical isolates observed in these studies may have evolved slightly, but we noted no significant change since then, suggesting that all the lineages were being continuously transmitted in this area.

In this study, the population density in every studied Fokontany was taken into account, increasing the accuracy of spatial clustering identification. The use of spoligotyping as a first screen is a relatively simple and inexpensive method. It allowed us to identify clusters that were overlooked using only spatial information. We thus plan to keep this strategy as a first-line detection of potential transmission areas. A possible improvement in our approach would be the use of methods providing both spoligotyping and resistance data such as TB-SPRINT [28]. This high-throughput assay tests for rifampicin and isoniazid resistance simultaneously with spoligotyping on Luminex. It could be a useful strategy in the near future to survey and prevent the spread of MDR-TB cases.

Another limit of this study was the short recruitment time (9 months). However, this recruitment period was sufficient to achieve a large sample size, and given the stability of the population, such duration should not have created too much bias. Some of the enrolled patients did not agree to give consent to participate in the study, those residing in Antananarivo Renivohitra consulting with DTCs outside Antananarivo, and those not consulting with a DTC had to be removed from the inclusion. Finally, we chose the patient residence to perform the spatial analysis although the patient residence is clearly not the unique site of possible TB transmission. Activity areas have been linked to transmission in other studies [11, 12]. We still chose to locate TB cases according to the residential Fokontany since people with most poor living condition and without work circulate most of their time in the same Fokontany in Antananarivo. A further investigation using the combined spatial methods on both working and residential areas could still be useful for TB epidemiological surveillance.

## Conclusion

Areas of high risk of TB transmission were suggested by the combination of *M. tuberculosis* spoligotyping and spatial analysis in the Urban District of Antananarivo. This method may be helpful for TB epidemiological surveillance in Madagascar and developing countries for guiding TB control strategies by the identification of priority target areas. Massive active diagnosis for children and susceptible persons can be suggested in these areas, as well as intense of TB awareness.

## Additional file

Additional file 1: Table S1. Distribution of the 88 spoligotypes obtained with the 394 typed patients. (DOCX 38 kb)

## References

[1] WHO (2015). Global Tuberculosis report 2015.

[2] WHO (2014). WHO Report 2014. Global tuberculosis control 2014.

[3] Kamerbeek J, Schouls L, Kolk A, van Agterveld M, van Soolingen D, Kuijper S (1997). Simultaneous detection and strain differentiation of *Mycobacterium tuberculosis* for diagnosis and epidemiology. J Clin Microbiol.

[4] Demay C, Liens B, Burguiere T, Hill V, Couvin D, Millet J (2012). SITVITWEB--a publicly available international multimarker database for studying *Mycobacterium tuberculosis* genetic diversity and molecular epidemiology. Infect Genet Evol.

[5] Brudey K, Driscoll JR, Rigouts L, Prodinger WM, Gori A, Al-Hajoj SA (2006). *Mycobacterium tuberculosis* complex genetic diversity: mining the fourth international spoligotyping database (SpolDB4) for classification, population genetics and epidemiology. BMC Microbiol.

[6] Comas I, Homolka S, Niemann S, Gagneux S. Genotyping of genetically monomorphic bacteria: DNA sequencing in *Mycobacterium tuberculosis* highlights the limitations of current methodologies. PLoS One. 2009;4(11):e7815. 10.1371/journal.pone.0007815 PMC2772813 19915672

[7] Stucki D, Ballif M, Egger M, Furrer H, Altpeter E, Battegay M, et al. Standard genotyping overestimates transmission of *Mycobacterium tuberculosis* among immigrants in a low incidence country. J Clin Microbiol. 2016;54(7):1862-70. 10.1128/JCM.00126-16 PMC4922098 27194683

[8] Supply P, Allix C, Lesjean S, Cardoso-Oelemann M, Rusch-Gerdes S, Willery E (2006). Proposal for standardization of optimized mycobacterial interspersed repetitive unit-variable-number tandem repeat typing of *Mycobacterium tuberculosis*. J Clin Microbiol.

[9] Small PM, Hopewell PC, Singh SP, Paz A, Parsonnet J, Ruston DC (1994). The epidemiology of tuberculosis in San Francisco-a population-based study using conventional and molecular methods. N Engl J Med.

[10] Moonan PK, Ghosh S, Oeltmann JE, Kammerer JS, Cowan LS, Navin TR (2012). Using genotyping and geospatial scanning to estimate recent *Mycobacterium tuberculosis* transmission, United States. Emerg Infect Dis.

[11] Izumi K, Ohkado A, Uchimura K, Murase Y, Tatsumi Y, Kayebeta A (2015). Detection of tuberculosis infection hotspots using activity spaces based spatial approach in an Urban Tokyo, from 2003 to 2011. PLoS One.

[12] Ribeiro FK, Pan W, Bertolde A, Vinhas SA, Peres RL, Riley L, et al. Genotypic and spatial analysis of *Mycobacterium tuberculosis* transmission in a high-incidence urban setting. Clin Infect Dis. 2015;61(5):758-66. 10.1093/cid/civ365 PMC4626752 25948063

[13] Gurjav U, Burneebaatar B, Narmandakh E, Tumenbayar O, Ochirbat B, Hill-Cawthorne GA (2015). Spatiotemporal evidence for cross-border spread of MDR-TB along the Trans-Siberian Railway line. Int J Tuberc Lung Dis.

[14] Kulldorff M, Nagarwalla N (1995). Spatial disease clusters: detection and inference. Stat Med.

[15] Randremanana RV, Sabatier P, Rakotomanana F, Randriamanantena A, Richard V (2009). Spatial clustering of pulmonary tuberculosis and impact of the care factors in Antananarivo City. Tropical Med Int Health.

[16] WHO (2014). Tuberculose: profil de pays/Madagascar.

[17] Rakotosamimanana S, Mandrosovololona V, Rakotonirina J, Ramamonjisoa J, Ranjalahy JR, Randremanana RV (2014). Spatial analysis of pulmonary tuberculosis in Antananarivo Madagascar: tuberculosis-related knowledge, attitude and practice. PLoS ONE.

[18] Randremanana RV, Richard V, Rakotomanana F, Sabatier P, Bicout DJ (2010). Bayesian mapping of pulmonary tuberculosis in Antananarivo, Madagascar. BMC Infect Dis.

[19] Ferdinand S, Sola C, Chanteau S, Ramarokoto H, Rasolonavalona T, Rasolofo-Razanamparany V (2005). A study of spoligotyping-defined *Mycobacterium tuberculosis* clades in relation to the origin of peopling and the demographic history in Madagascar. Infect Genet Evol.

[20] Razanamparany VR, Ménard D, Aurégan G, Gicquel B, Chanteau S (2002). Extrapulmonary and pulmonary tuberculosis in Antananarivo (Madagascar): high clustering rate in female patients. J Clin Microbiol.

[21] Rasolofo-Razanamparany V, Auregan G, Ratsirahonana O, Raharimanana R, Ramarokoto H, Gicquel B (1994). Genetic polymorphism of *M. tuberculosis* strains in Antananaviro. Arch Inst Pasteur Madagascar.

[22] Wachsberger J-M. Les quartiers pauvres à Antananarivo. Autrepart. 2009(3):117–37. http://www.cairn.info/revue-autrepart-2009-3-page-117.htm. Accessed 3 June.

[23] Zhang J, Abadia E, Refregier G, Tafaj S, Boschiroli ML, Guillard B (2010). *Mycobacterium tuberculosis* complex CRISPR genotyping: improving efficiency, throughput and discriminative power of ‘spoligotyping’ with new spacers and a microbead-based hybridization assay. J Med Microbiol.

[24] Coll F, McNerney R, Guerra-Assunçào JA, Glynn JR, Perdigào J, Viveiros M (2014). A robust SNP barcode for typing *Mycobacterium tuberculosis* complex strains. Nat Commun.

[25] Coll F, Preston M, Guerra-Assunçào JA, Hill-Cawthorn G, Harris D, Perdigào J (2014). PolyTB: a genomic variation map for *Mycobacterium tuberculosis*. Tuberculosis.

[26] Kulldorff M (1997). A spatial scan statistic. Communications in statistics.

[27] Rakotosamimanana N, Raharimanga V, Andriamandimby SF, Soares JL, Doherty TM, Ratsitorahina M (2010). Variation in gamma interferon responses to different infecting strains of *Mycobacterium tuberculosis* in acid-fast bacillus smear-positive patients and household contacts in Antananarivo, Madagascar. Clin Vaccine Immunol.

[28] Gomgnimbou MK, Hernandez-Neuta I, Panaiotov S, Bachiyska E, Palomino JC, Martin A (2013). Tuberculosis-spoligo-rifampin-isoniazid typing: an all-in-one assay technique for surveillance and control of multidrug-resistant tuberculosis on Luminex devices. J Clin Microbiol.

