# Supplementary material for: Assessment of tuberculosis spatial hotspot areas in Antananarivo, Madagascar, by combining spatial analysis and genotyping
Source: BMC Infect Dis. 2017 Aug 14;17:562. doi: 10.1186/s12879-017-2653-9 (PMC5557477; doi:10.1186/s12879-017-2653-9)
Supplement: Additional file 1: Table S1. — Distribution of the 88 spoligotypes obtained with the 394 typed patients. (DOCX 38 kb) [file 12879_2017_2653_MOESM1_ESM.docx]

**Additional file**

**Table S1: Distribution of the 88 spoligotypes obtained with the 394 typed patients.**

|  | Spoligotypes | SIT | Sublineages  (Demay et al  2012) | Lineage  (Coll et al. 2014) | number |
| --- | --- | --- | --- | --- | --- |
| 1 |  | 523 | U | U | 1 |
| 2 |  | 100 | MANU1 | MANU | 1 |
| 3 |  | 1196 | U | U | 2 |
| 4 |  | 53 | T1 | L4 | 18 |
| 5 |  | 52 | T2 | L4 | 2 |
| 6 |  | 78 | T2 | L4 | 18 |
| 7 |  | 245 | T1 | L4 | 2 |
| 8 |  | 65 | T1 | L4 | 2 |
| 9 |  | 242 | T1 | L4 | 1 |
| 10 |  | IND 1 | MANU1 | MANU | 2 |
| 11 |  | IND 10 | U | U | 1 |
| 12 |  | 50 | H3 | L4 | 7 |
| 13 |  | IND 21 | U | U | 1 |
| 14 |  | 237 | U | U | 1 |
| 15 |  | 47 | H1 | L4 | 5 |
| 16 |  | 62 | H1 | L4 | 9 |
| 17 |  | 46 | U | U | 3 |
| 18 |  | 373 | T1 | L4 | 1 |
| 19 |  | IND 28 | T IND | L4 | 1 |
| 20 |  | 44 | T5 | L4 | 2 |
| 21 |  | 1580 | T1 | L4 | 1 |
| 22 |  | 61 | LAM10_CAM | L4 | 7 |
| 23 |  | IND 29 | MANU2 | MANU | 1 |
| 24 |  | IND 30 | U | U | 1 |
| 25 |  | 86 | T1 | L4 | 39 |
| 26 |  | 283 | H1 | L4 | 1 |
| 27 |  | 42 | LAM9 | L4 | 2 |
| 28 |  | 60 | LAM4 | L4 | 1 |
| 29 |  | 162 | LAM9 | L4 | 2 |
| 30 |  | IND 31 | LAM IND | L4 | 1 |
| 31 |  | 59 | LAM11_ZWE | L4 | 12 |
| 32 |  | 74 | T1 | L4 | 9 |
| 33 |  | 58 | T5_MAD2 | L4 | 1 |
| 34 |  | 1328 | H1 | L4 | 2 |
| 35 |  | 336 | X1 | L4 | 4 |
| 36 |  | 1678 | H3 | L4 | 1 |
| 37 |  | IND 2 | T IND | L4 | 3 |
| 38 |  | 37 | T3 | L4 | 1 |
| 39 |  | 73 | T3 | L4 | 2 |
| 40 |  | IND 3 | T IND | L4 | 2 |
| 41 |  | 1214 | T1 | L4 | 5 |
| 42 |  | IND 32 | T IND | L4 | 1 |
| 43 |  | 136 | T1 | L4 | 1 |
| 44 |  | IND 11 | T IND | L4 | 1 |
| 45 |  | 149 | T3_ETH | L4 | 2 |
| 46 |  | 1129 | T1 | L4 | 1 |
| 47 |  | 34 | S | L4 | 7 |
| 48 |  | 784 | T2 | L4 | 1 |
| 49 |  | 156 | T1 | L4 | 38 |
| 50 |  | 33 | LAM3 | L4 | 2 |
| 51 |  | IND 12 | T IND | L4 | 1 |
| 52 |  | IND 13 | T IND | L4 | 1 |
| 53 |  | 1223 | T1 | L4 | 1 |
| 54 |  | IND 8 | T1 | L4 | 1 |
| 55 |  | 31 | T1 | L4 | 3 |
| 56 |  | IND 14 | T IND | L4 | 1 |
| 57 |  | 280 | T1_RUS2 | L4 | 1 |
| 58 |  | 99 | H3 | L4 | 3 |
| 59 |  | IND 15 | H3 | L4 | 1 |
| 60 |  | IND 16 | MANU2 | MANU | 1 |
| 61 |  | IND 17 | T IND | L4 | 1 |
| 62 |  | 1361 | T IND | L4 | 3 |
| 63 |  | 26 | CAS1_DELHI | L3 | 3 |
| 64 |  | 25 | CAS1_DELHI | L3 | 1 |
| 65 |  | IND 9 | CAS1_KILI | L3 | 1 |
| 66 |  | 21 | CAS1_KILI | L3 | 35 |
| 67 |  | 196 | T1 | L4 | 1 |
| 68 |  | 1332 | T2 | L4 | 1 |
| 69 |  | IND 18 | MANU2 | MANU | 1 |
| 70 |  | IND 19 | U | L4 | 1 |
| 71 |  | 1673 | BOVIS | bovis | 1 |
| 72 |  | IND 4 | MANU1 | MANU | 2 |
| 73 |  | IND 20 | CAS1_KILI | L3 | 1 |
| 74 |  | IND 5 | EAI IND | L1 | 2 |
| 75 |  | 109 | EAI8_MDG | L1 | 37 |
| 76 |  | IND 22 | EAI8_MDG | L1I | 1 |
| 77 |  | IND 23 | EAI8_MDG | L1 | 1 |
| 78 |  | 10 | EAI8_MDG | L1 | 3 |
| 79 |  | IND 24 | EAI8_MDG | L1 | 1 |
| 80 |  | IND 6 | EAI8_MDG | L1 | 2 |
| 81 |  | IND 25 | EAI IND | L1 | 1 |
| 82 |  | 76 | EAI8_MDG | L1 | 1 |
| 83 |  | IND 26 | EAI8_MDG | L1 | 1 |
| 84 |  | 376 | LAM3 | L1 | 1 |
| 85 |  | 1525 | LAM3 | L1 | 3 |
| 86 |  | IND 27 | LAM9 | L1 | 1 |
| 87 |  | IND 7 | H IND | L1 | 2 |
| 88 |  | 1 | BEIJING | L2 | 39 |
